# MicroRNA-874 prohibits the proliferation and invasion of retinoblastoma cells by directly targeting metadherin

**DOI:** 10.3892/mmr.2022.12900

**Published:** 2022-11-21

**Authors:** Yongfeng Zhang, Xueqin Wang, Yuehua Zhao

Mol Med Rep 18: 3099–3105, 2018; DOI: 10.3892/mmr.2018.9295

Following the publication of this paper, it was drawn to the Editors’ attention by a concerned reader that certain of the data showing cell invasion assay experiments in Figs. 2C, 4D and 5D were strikingly similar to data that had appeared in different form in other articles by different authors. Owing to the fact that the contentious data in the above article had already been published elsewhere, or were already under consideration for publication, prior to its submission to *Molecular Medicine Reports*, the Editor has decided that this paper should be retracted from the Journal. The authors were asked for an explanation to account for these concerns, but the Editorial Office did not receive a reply. The Editor apologizes to the readership for any inconvenience caused.

